# Sibling recurrence risk ratio analysis of the metabolic syndrome and its components over time

**DOI:** 10.1186/1471-2156-4-S1-S33

**Published:** 2003-12-31

**Authors:** Wei J Chen, Pi-Hua Liu, Yen-Yi Ho, Kuo-Liong Chien, Min-Tzu Lo, Wei-Liang Shih, Yu-Chun Yen, Wen-Chung Lee

**Affiliations:** 1 Institute of Epidemiology, College of Public Health, National Taiwan University, Taipei, Taiwan; 2 Institute of Preventive Medicine, College of Public Health, National Taiwan University, Taipei, Taiwan

## Abstract

**Background:**

The purpose of this study was to estimate both cross-sectional sibling recurrence risk ratio (λ_*s*_) and lifetime λ_*s *_for the metabolic syndrome and its individual components over time among sibships in the prospectively followed-up cohorts provided by the Genetic Analysis Workshop 13. Five measures included in the operational criteria of the metabolic syndrome by the Adult Treatment Panel III were examined. A method for estimating sibling recurrence risk with correction for complete ascertainment was used to estimate the numerator, and the prevalence in the whole cohort was used as the denominator of λ_*s*_.

**Results:**

Considerable variability in the λ_*s *_was found in terms of different time-points for the cross-sectional definition, the times of fulfilling the criterion for lifetime definition, and different components. Obesity and hyperglycemia had the highest cross-sectional λ_*s *_of the five components. Both components also had the largest slopes in the linear trend of the lifetime λ_*s*_. However, the magnitudes of the lifetime λ_*s *_were similar to that of the mean cross-sectional λ_*s*_, which were <2. The results of nonparametric linkage analysis showed only suggestive evidence of linkage between one marker and lifetime diagnosis of low high-density lipoprotein cholesterol and metabolic syndrome, respectively.

**Conclusion:**

The λ_*s *_of the metabolic syndrome and its components varies substantially across time, and the λ_*s *_of lifetime diagnosis was not necessarily larger than that of a cross-sectional diagnosis. The magnitude of λ_*s *_does not predict well the maximum LOD score of linkage analysis.

## Background

The metabolic syndrome is a clustering of diabetic and cardiovascular risk factors, including obesity, hypertriglyceridemia, low high-density lipoprotein (HDL) cholesterol, hypertension, and glucose intolerance [1]. It predicts both diabetes and cardiovascular diseases, and indicates the existence of a common underlying mechanism linking these two disorders. A recent study of twins found that the concordance rates for glucose concentration, overall obesity, and HDL-cholesterol were significantly higher among monozygotic twins than dizygotic twins [2]. However, the heritability estimates for waist-to-hip ratio, fasting insulin, and triglycerides were found to be low. Another study employing factor analysis of the change in the features of the metabolic syndrome over time revealed that change in overall obesity, as measured by the body mass index (BMI), is central to all derived factors [3]. These findings indicate that the components of the metabolic syndrome may have different contributions from genes and environment, and certain component may be central to the whole syndrome.

In assessing the familial aggregation of a disease or physiological trait, the sibling recurrence risk ratio, λ_*s*_, is a commonly used index [4]. Under a multilocus multiplicative model, λ_*s *_is proportional to the power of an affected-relative-pair genetic linkage analysis [5]. Recent studies have revealed that λ_*s *_is influenced by many factors, including the ascertainment process and overreporting [6], as well as allele frequency and mode of inheritance [7]. Thus, λ_*s *_by itself does not provide a reliable parameter for estimating the statistical power of a proposed linkage study. Nevertheless, within a range of low magnitudes of genetic effect, which probably is true for many susceptibility genes of complex diseases, the relationship between λ_*s *_and genotype relative risk is relatively predictable regardless of underlying genetic models [7]. Furthermore, there are methods to estimate λ_*s *_with correction for ascertainment [8]. Therefore, an accurate estimate of λ_*s *_can serve as an initial assessment of the genetic effect conferred to a trait under study.

So far λ_*s *_has been estimated exclusively on the basis of cross-sectional assessment of phenotypes. When a disease has a variable age at onset and the phenotype is repeatedly measured, it is not known whether the λ_*s *_is stable over time. In this study, we estimated both cross-sectional λ_*s *_and lifetime λ_*s *_for the metabolic syndrome and its individual components over time among sibships in a prospectively followed-up cohort. We hypothesized that a component with a higher λ_*s *_is more likely to be the central genetic contribution to the syndrome. The distributions of various definitions of λ_*s *_over time for the metabolic syndrome and its individual components were compared. In addition, genome-wide scans were performed for both cross-sectional and lifetime phenotype definitions for the metabolic syndrome and its individual components.

## Methods

### Subjects

Participants were from Cohort 1 and Cohort 2 of the Framingham Heart Study data set provided by the Genetic Analysis Workshop 13 (GAW13). In Cohort 1, 1231 individuals (583 males and 630 females) with a mean baseline age of 41.5 years (ranging from 29.0 to 62.0) were included. Cohort 2 included 1672 individuals (826 males and 846 females) with a mean baseline age of 32.7 years (ranging from 5.0 to 64.0). Some participants in the two cohorts were from the same pedigree, and in total 330 pedigrees were identified. Among these pedigrees, 1702 individuals were further genotyped for genomic scanning.

### Measures

In this study, we considered five measures that are included in the operational criteria for the metabolic syndrome as defined by the Adult Treatment Panel III (ATP III) [9]. Because the data on waist circumference, which measures abdominal obesity, were not provided in the Framingham Heart Study data set, we instead employed overall obesity as measured by a BMI > 30 kg/m^2^. The cut-off points of hypertension in the Framingham Heart Study data set were slightly more stringent than those recommended in the ATP III criteria (≥ 130 / ≥ 85 mm Hg). The specific criteria adopted in this study were as follows: 1) Blood pressure: systolic blood pressure (SBP) ≥ 140 mm Hg or diastolic blood pressure (DBP) ≥ 90 mm Hg, or under anti-hypertension treatment; 2) Obesity: BMI > 30 kg/m^2^; 3) Fasting glucose: ≥ 110 mg/dl; 4) Triglycerides: ≥ 150 mg/dl; and 5) HDL cholesterol: < 40 mg/dl (men) or < 50 mg/dl (women). The diagnosis of the metabolic syndrome was made when three or more of the criteria were met. Phenotypic data provided in the GAW13 included those for the first 40 years of follow-up in the Cohort 1 (Exams 1 through 21) and those of the first 20 years of follow-up in the Cohort 2 (Exams 1 through 5).

### Statistical analysis

Sibling recurrence risk ratio (λ_*s*_) is defined as sibling recurrence risk (*K*_*s*_) divided by the population prevalence (*K*_*p*_). Since the original pedigrees were not ascertained via any particular phenotype considered in the present study, the determination of proband status is problematic. Under this circumstance, we considered every diseased individual as a proband and employed the method for estimating sibling recurrence risk (*K*_*s*_) when proband status is unknown as proposed by Olson and Cordell [8]:



where *n*_*s*(*a*) _is the number of sibships of size *s *with *a *affecteds. This estimator of *K*_*s *_has been shown to be unbiased and consistent when the ascertainment is complete [8], which is the case for this study. The denominator of λ_*s*_, the population prevalence, was estimated from the whole cohort. Since the age range of Cohort 2 was wide, we limited the analysis of Cohort 2 to those aged between 30 and 60 years old.

We estimated cross-sectional λ_*s *_for each exam and then calculated the standard deviation and the range of the estimated λ_*s *_across the exams. Furthermore, we defined a lifetime λ_*s*,*e*≥*t *_as the condition that an individual had ever had the *episode *fulfilling the criterion at least *t *times during the whole study period. As *t *increases, the person is supposed to have higher probability of carry the genetic susceptibility of the phenotype. In assessing the lifetime λ_*s *_for the metabolic syndrome, any particular criterion was considered fulfilled if the individual had ever any measures above the threshold during lifetime, and a positive diagnosis was given if three or more criteria were met in this way.

For each component and the metabolic syndrome per se, we then performed nonparametric linkage analyses for the pedigrees using GENEHUNTER [10] with the option of all affected pairs in a pedigree. Four kinds of phenotype definition were employed: cross-sectional diagnosis, lifetime diagnosis with episode ≥ 1, lifetime diagnosis with episode ≥ 2, and lifetime diagnosis with episode ≥ 3. For the cross-sectional diagnosis, we chose the data from Exam 11 for Cohort 1 members and Exam 1 for Cohort 2 members because the two test points were chronologically close to each other. However, for Cohort 1 members, the height was from Exam 10 and the glucose level was from Exam 12 because no relevant data were available in Exam 11. The genotyping was done in 1702 individuals using 399 microsatellite markers on the 22 autosomal chromosomes. The allele frequencies of the markers and the sex-specific genetic maps were provided by the GAW13. The number of affected individuals for the four phenotype definitions (cross-sectional, lifetime ≥ 1, lifetime ≥ 2, lifetime ≥ 3) varied as follows: (255, 898, 713, 545) for hypertension, (185, 512, 384, 304) for obesity, (341, 597, 314, 182) for hyperglycemia, (228, 656, 348, 208) for hypertriglyceridemia, (522, 1046, 700, 482) for low HDL cholesterol, and (147, 699) for the metabolic syndrome (only cross-sectional and lifetime ≥ 1 were applicable).

## Results

**Figure 1:**
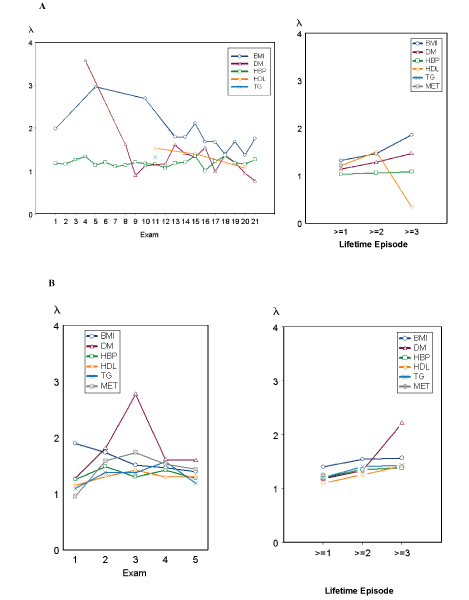
The trends of sibling recurrence risk ratios λ_*s *_of individual components of the metabolic syndrome and the syndrome per se in Cohort 1 (A) and Cohort 2 (B).

For the cross-sectional λ_*s *_in both cohorts, there were substantial variations across exams for hyperglycemia and obesity, while the values of λ_*s *_for hypertension and low HDL were relatively stable (Figure 1). Meanwhile, an increasing trend with the number of episode was noted for the lifetime λ_*s *_except those of hypertension and low HDL in Cohort 1. Comparing the mean value of cross-sectional λ_*s *_over time in Cohort 1, obesity had the highest mean, while the remaining components had a mean λ_*s *_less than 1.5 (Table 1). In terms of the SD of the cross-sectional λ_*s*_, there were considerable differences among different components, ranging from 0.14 (hypertension) to 0.68 (hyperglycemia). For the lifetime λ_*s *_in Cohort 1, a linear trend of increase with the number of episode was noted for obesity and hyperglycemia. The lifetime λ_*s *_of low HDL had a sudden drop at λ_*s*,*e*≥3_, probably due to small number of participants meeting the threshold, whereas the three lifetime λ_*s *_of hypertension were very similar to one another.

For the Cohort 2, the highest mean of cross-sectional λ_*s *_for individual components of the metabolic syndrome was that for hyperglycemia, followed by obesity (Table 1). For the remaining three components, the mean λ_*s *_was below 1.5. A linear trend of increase with the number of episode among the three types of lifetime λ_*s *_could be seen in all components, with hyperglycemia having the largest increase. However, similar to the pattern observed in Cohort 1, the lifetime definition of λ_*s *_led to a decrease over the cross-sectional definition in most cases. The λ_*s*_of the metabolic syndrome, regardless of cross-sectional or lifetime, seemed to fall between those of its components in both Cohort 1 and Cohort 2.

The markers that had a maximum LOD score ≥ 1.5 in the genome-wide scan is displayed in Table 2. For the cross-sectional diagnosis, only hypertriglyceridemia and low HDL cholesterol had such markers, while for the lifetime diagnosis each component and the metabolic syndrome had markers with LOD score ≥ 1.5, with the lifetime diagnosis with episode ≥ 1 having the most such markers. The same marker did not consistently appear in all phenotype definitions. Some markers appeared in more than one trait under the same type of phenotype definition, such as C12g3 in obesity, low HDL, and the metabolic syndrome, and C17g2 in hypertension and hypertriglyceridemia. Overall, suggestive evidence of linkage (LOD ≥ 2.2) was found only for the lifetime diagnosis with episode ≥ 1 of low HDL and the metabolic syndrome.

## Discussion

The results of the λ_*s *_analyses demonstrated that there is a modest to moderate magnitude of familial aggregation for the metabolic syndrome and its components. However, considerable variability in λ_*s *_was found in terms of different time-points for the cross-sectional definition, the times of fulfilling the criterion for the lifetime definition, and different components of the metabolic syndrome.

The variability of cross-sectional λ_*s *_over time is quite different among the components of the metabolic syndrome, with hypertension having the smallest SD and hyperglycemia having the largest SD in both Cohort 1 and Cohort 2. This finding highlights the instability of a single time-point estimate of λ_*s *_for certain phenotypic measures. Several reasons may account for this. First, the distribution of the original phenotypic measures was skewed for the majority of components and was not bimodal in shape. The arbitrary threshold used by the ATP III criteria might lead to fluctuation over time in prevalence and the subsequent estimates of λ_*s*_. Second, the number of siblings eligible for *K*_*s *_estimation became very small when the prevalence was low, e.g., around 30 for hyperglycemia in Cohort 1.

With our definitions of lifetime λ_*s*,*e*≥*t*_, it is expected that the more times a person meets the criteria, the higher probability that the individual carries the susceptibility genotype(s). Indeed, a linear trend in lifetime λ_*s *_was noted in the majority of the components of the metabolic syndrome, especially hyperglycemia. However, the value of the lifetime λ_*s*,*e*≥3 _was not necessarily greater than the mean cross-sectional λ_*s *_of its counterpart, with some even lower than the latter (e.g., obesity). This may further call into question the use of cross-sectional λ_*s*_.

Comparing the one-time λ_*s *_of the components of the metabolic syndrome, obesity and hyperglycemia stand out as the highest components for both Cohort 1 and Cohort 2. Both components also had the largest slope in the linear trend of the lifetime λ_*s*_. Thus, both obesity and hyperglycemia might be the central components of the metabolic syndrome in terms of familiality. This is consistent with the findings in twin analysis [2] and longitudinal factor analysis [3] of the metabolic syndrome.

Despite the evidence supporting the familial aggregation of the metabolic syndrome and its components, the modest to moderate magnitude of the λ_*s *_(its mean not greater than 2) found in this study implies that very large samples of affected relative pairs are needed for the detection of definitive genetic linkage [5,7]. This indeed is the result for our nonparametric linkage analysis of the metabolic syndrome and its components, respectively. Although it appeared that there was no relation between the magnitude of λ_*s *_and the number of markers with LOD score ≥ 1.5, the widely varying number of affected individuals for each component might account in part for this. Nevertheless, the repeated appearances of some markers across different traits indicate that these traits might indeed share certain common genetic susceptibility.

## Figures and Tables

**Table 1:** The sibling recurrence risk ratios (λ_*s*_) of individual components of the metabolic syndrome and the syndrome per se in Cohort 1 and Cohort 2.

	Cross-sectional λ_*s*_			Lifetime λ_*s*_		
		
Variable	*n*	Mean (SD)	Range	λ_*s*,*e*≥1_	λ_*s*,*e*≥2_	λ_*s*,*e*≥3_
Cohort 1						
Hypertension	21	1.22 (0.14)	1.11–1.72	1.03	1.04	1.07
Obesity (high BMI)	12	1.91 (0.48)	1.37–2.95	1.32	1.46	1.86
Hyperglycemia	14	1.39 (0.68)	0.77–3.57	1.14	1.28	1.47
Hypertriglyceridemia	1^A^	1.32	-	1.28	-	-
Low HDL cholesterol	3	1.34 (0.23)	1.07–1.52	1.21	1.50	0.34
Metabolic syndrome		-	-	1.21	-	-
Cohort 2^B^						
Hypertension	5	1.34 (0.10)	1.25–1.48	1.20	1.35	1.38
Obesity (high BMI)	5	1.60 (0.21)	1.39–1.89	1.40	1.54	1.56
Hyperglycemia	5	1.81 (0.57)	1.27–2.77	1.18	1.32	2.22
Hypertriglyceridemia	5	1.32 (0.19)	1.09–1.56	1.20	1.41	1.43
Low HDL cholesterol	5	1.30 (0.10)	1.15–1.42	1.10	1.25	1.42
Metabolic syndrome	5	1.45 (0.30)	0.95–1.74	1.25	-	-

^A^The other two measures of triglycerides led to too few subjects for the estimation. ^B^Limited to subjects aged 30–60 years.

**Table 2:** Results of nonparametric linkage analysis of genome-wide scanning with LOD score ≥ 1.5 (if two or more adjacent markers, only the one with the highest score listed).

Trait	Cross-Sectional Diagnosis Marker (LOD)	Lifetime Diagnosis		
		
		Episode ≥ 1 Marker (LOD)	Episode ≥ 2 Marker (LOD)	Episode ≥ 3 Marker (LOD)
Hypertension	-	C1g32 (1.6)	C1g32 (1.6)	C1g32 (1.6)
		C17g2 (2.1)	C9g11 (1.7)	C9g11 (1.7)
		C22g1 (1.5)	C22g1 (1.5)	C22g1 (1.5)
Obesity (high BMI)	-	-	C8g10 (1.5)	C8g10 (1.5)
	-	C12g3 (1.5)	-	-
	-	C19g11 (1.5)	-	-
Hyperglycemia	-	C3g26 (1.5)	-	-
	-	C8g10 (1.5)	-	-
	-	C10g8 (1.5)	-	-
Hypertriglyceridemia	-	C6g8 (1.8)	-	-
	C10g12 (1.5)	C10g12 (1.6)	-	-
	-	C13g9 (1.5)	C13g9 (1.5)	C13g9 (1.5)
	-	C17g2 (1.9)	-	-
	C22g7 (1.5)	C22g7 (1.5)	C22g7 (1.5)	-
Low HDL cholesterol		C9g7 (2.0)	-	-
	C10g11 (1.5)	-	C10g12 (1.8)	C10g12 (1.6)
	-	C12g3 (1.8)	-	-
	-	-	C13g8 (1.5)	C13g8 (1.5)
	-	C16g3 (1.9)	-	-
	-	-	C18g9 (1.8)	-
	C19g3 (1.8)	C19g3 (2.4)	-	-
	-	-	C22g7 (1.8)	-
Metabolic syndrome	-	C9g12 (1.5)	NA^A^	NA
	-	C10g2 (1.5)		
	-	C12g3 (1.5)		
	-	C19g8 (2.0)		
	-	C22g7 (2.2)		

^A^Not done because many subjects did not have complete information on all five components.
